# “Water running in my chest”: Delayed spontaneous rupture of an aortocoronary saphenous vein graft aneurysm

**DOI:** 10.1002/ccr3.4024

**Published:** 2021-03-04

**Authors:** Eric Gnall, Manju Bengaluru Jayanna, Timothy Shapiro, Muhammad Abdulbasit, Maria Elena Drosou, Tariq Ahmad

**Affiliations:** ^1^ Department of Cardiology, Lankenau Medical Center Wynnewood PA USA; ^2^ Division of Nephrology Department of Medicine Penn State Health Milton S. Hershey Medical Center Hershey PA USA; ^3^ Department of Internal Medicine Lankenau Medical Center Wynnewood PA USA

**Keywords:** coronary artery bypass graft, intravascular ultrasound., obtuse marginal, saphenous vein graft, saphenous vein graft aneurysmal dilation, transesophageal echocardiogram

## Abstract

Saphenous vein graft aneurysm is an uncommon condition and knowledge about its natural history, and a multi‐specialty heart team approach is of utmost importance for better clinical outcomes. This case highlights importance of percutaneous intervention as a viable therapeutic option in the case of saphenous vein graft aneurysms.

## Graphical abstract

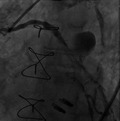

## 1 CASE DESCRIPTION

Saphenous vein graft aneurysmal dilation (SVGA), usually a delayed complication of atherosclerotic degeneration of the graft, is a rare but potentially fatal condition. We present a case of spontaneous SVGA hemorrhage in a 25‐year‐old graft, treated with a covered stent while preserving blood flow to a large myocardial territory.

**FIGURE 1:**
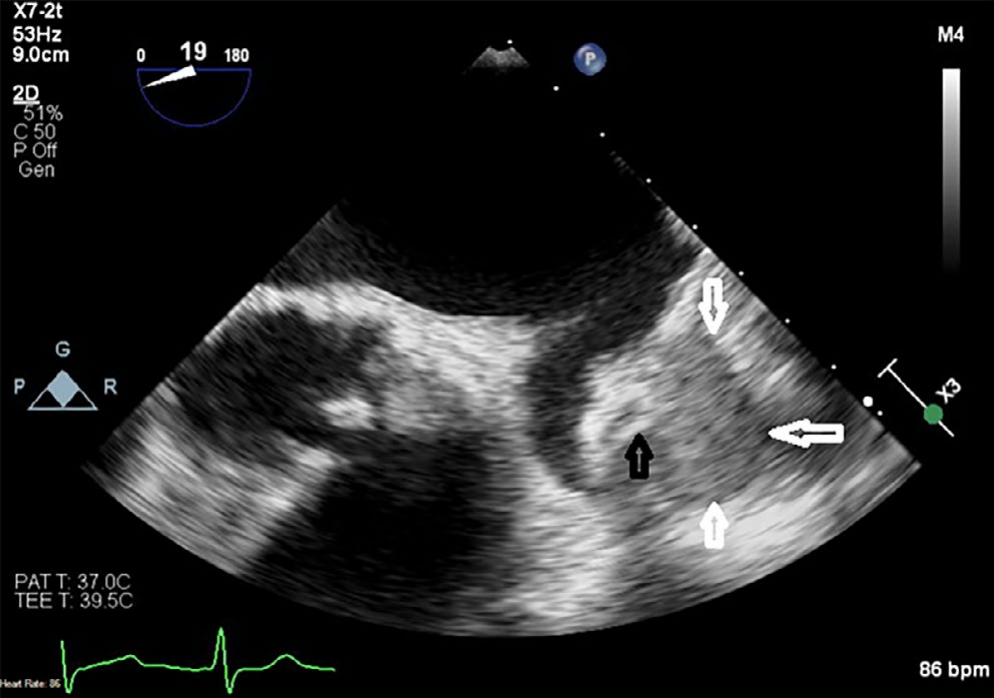
Transesophageal echocardiogram showing a 2.8 × 3.6 cm mass compressing left atrial appendage (white arrows) and saphenous vein graft stent (black arrow)

**FIGURE 2:**
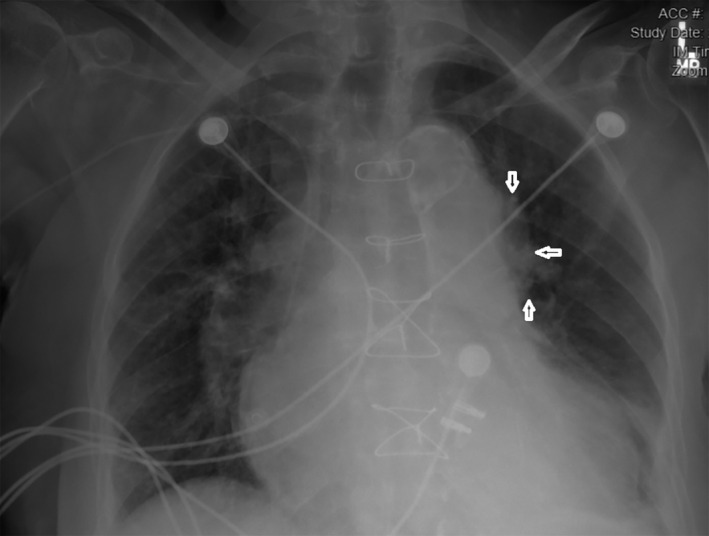
Chest x‐ray showing a left hilar mass (black arrows)

**FIGURE 3:**
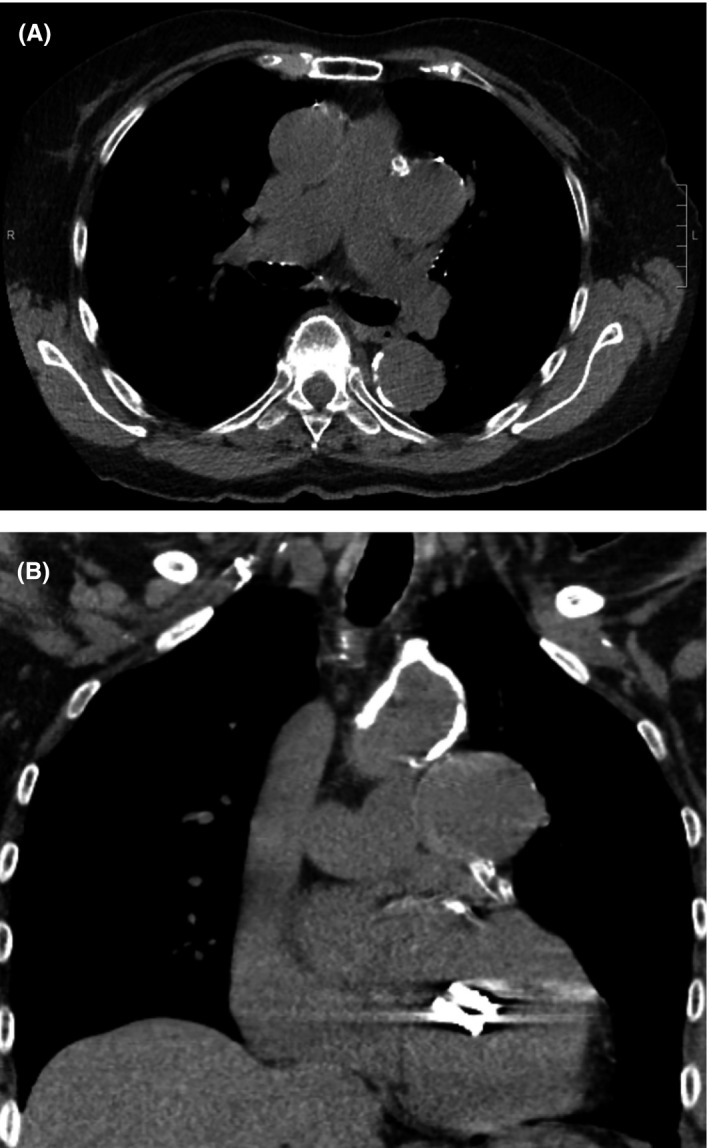
A, Noncontrast CT chest(transverse) showing a 44 × 39 mm aneurysmal dilation of SVG to OM. B: Noncontrast CT chest(coronal) showing a 44 × 39 mm aneurysmal dilation of SVG to OM

**FIGURE 4:**
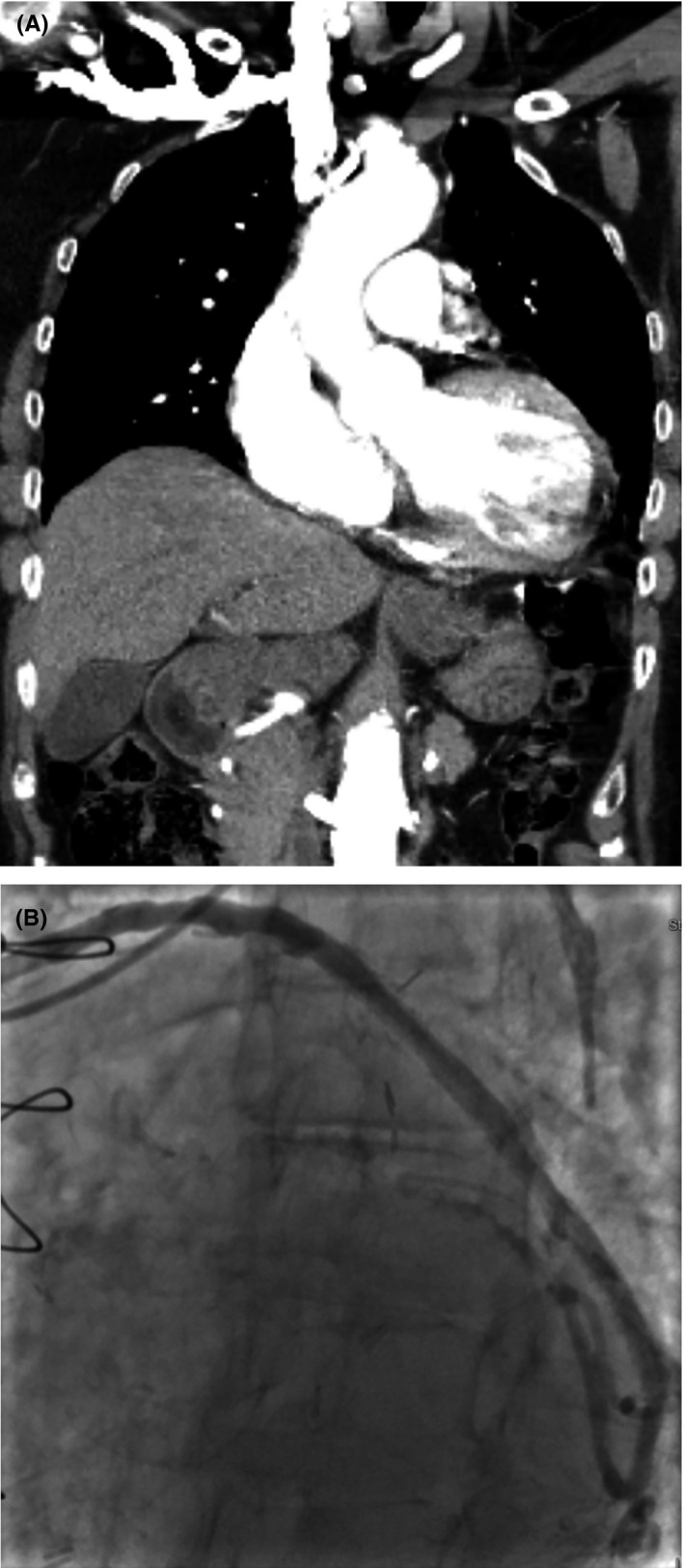
A, Contrast CT angiogram showing no aneurysmal dilation 6 y ago. B: Coronary angiogram of SVG to OM showing no significant aneurysmal dilation 6 y ago

An 82‐year‐old woman with a history of coronary artery bypass graft (CABG) (Left internal mammary artery to left anterior descending, saphenous vein graft (SVG) to posterior descending artery and sequential SVG to obtuse marginal(OM) 2 and 3) in 1995, prior drug‐eluting stent placement in SVG to OM and mitral regurgitation treated with percutaneous edge‐to‐edge repair 6 years ago, and an episode of paroxysmal atrial fibrillation 6 months ago who was found to have an extra‐cardiac echo‐reflective 2.8 × 3.6 cm mass compressing left atrial appendage on precardioversion transesophageal echocardiogram (TEE) (Figure 1). Chest x‐ray showed a left hilar mass (Figure 2). Noncontrast CT chest (Figure 3A, 3B) was performed due to underlying chronic kidney disease stage III‐IV, demonstrated a 44 × 39 mm aneurysmal dilation of SVG to OM. Six year ago, CT chest (Figure 4A) and coronary angiography (Figure 4B) did not show any SVGA. The decision was made to perform follow‐up imaging which was delayed due to ongoing COVID‐related restrictions. She presented to the hospital after she noticed sudden symptoms of “water running in my chest” followed by chest pain below her left breast and lightheadedness. The differential diagnosis included SVGA rupture, acute coronary syndrome, pulmonary embolism, and aortic dissection.

**FIGURE 5:**
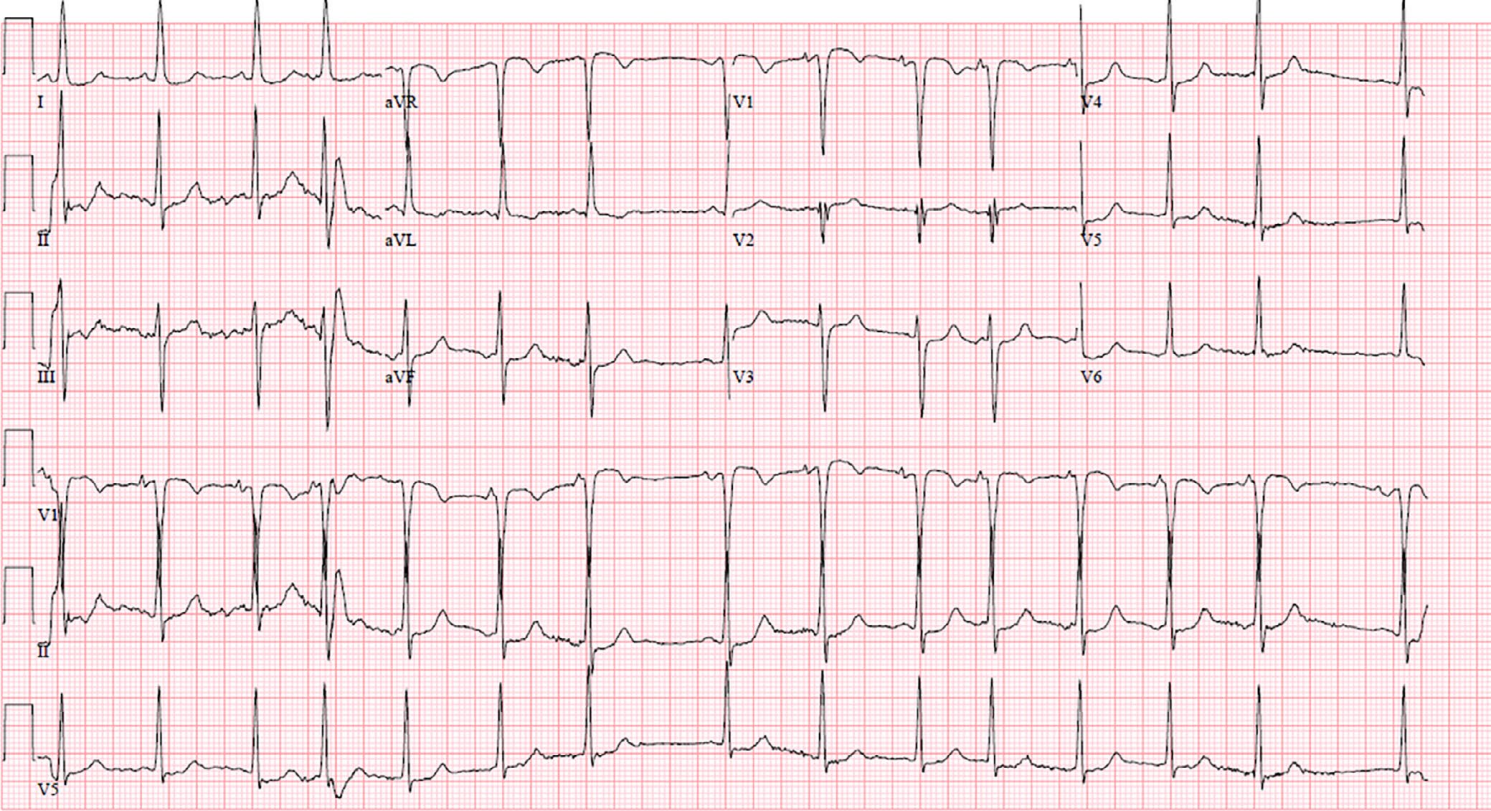
EKG on presentation to ER showing Sinus rhythm, Premature Atrial complexes

**FIGURE 6:**
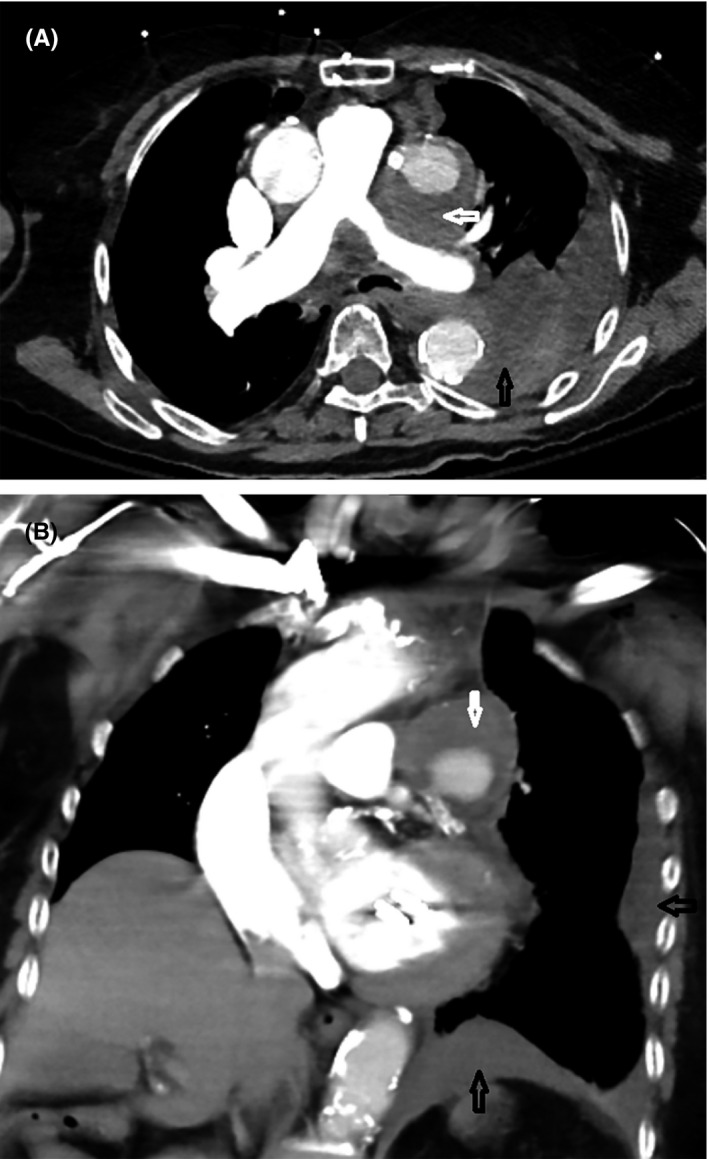
A, Contrast CT angiogram of chest (transverse) showing a 45 × 40 mm pseudoaneurysm of SVG to OM with large clot burden (white arrow) along with large left‐sided hemothorax (black arrow). B, Contrast CT angiogram of chest (coronal) showing a 45 × 40 mm pseudoaneurysm of SVG to OM with large clot burden (white arrow) along with large left‐sided hemothorax (black arrow)

In the ER, she was found to be hypotensive (BP 80/52 mm Hg) with a hemoglobin of 10.4 g/dL from 13 g/dL 6 months earlier. Electrocardiogram was unremarkable. (Figure 5). Contrast CT angiogram of the chest demonstrated a 45 x 40 mm pseudoaneurysm of the SVG to OM along with large left‐sided hemothorax (Figure 6A, B). She was emergently taken to the cardiac catheterization laboratory.

**FIGURE 7:**
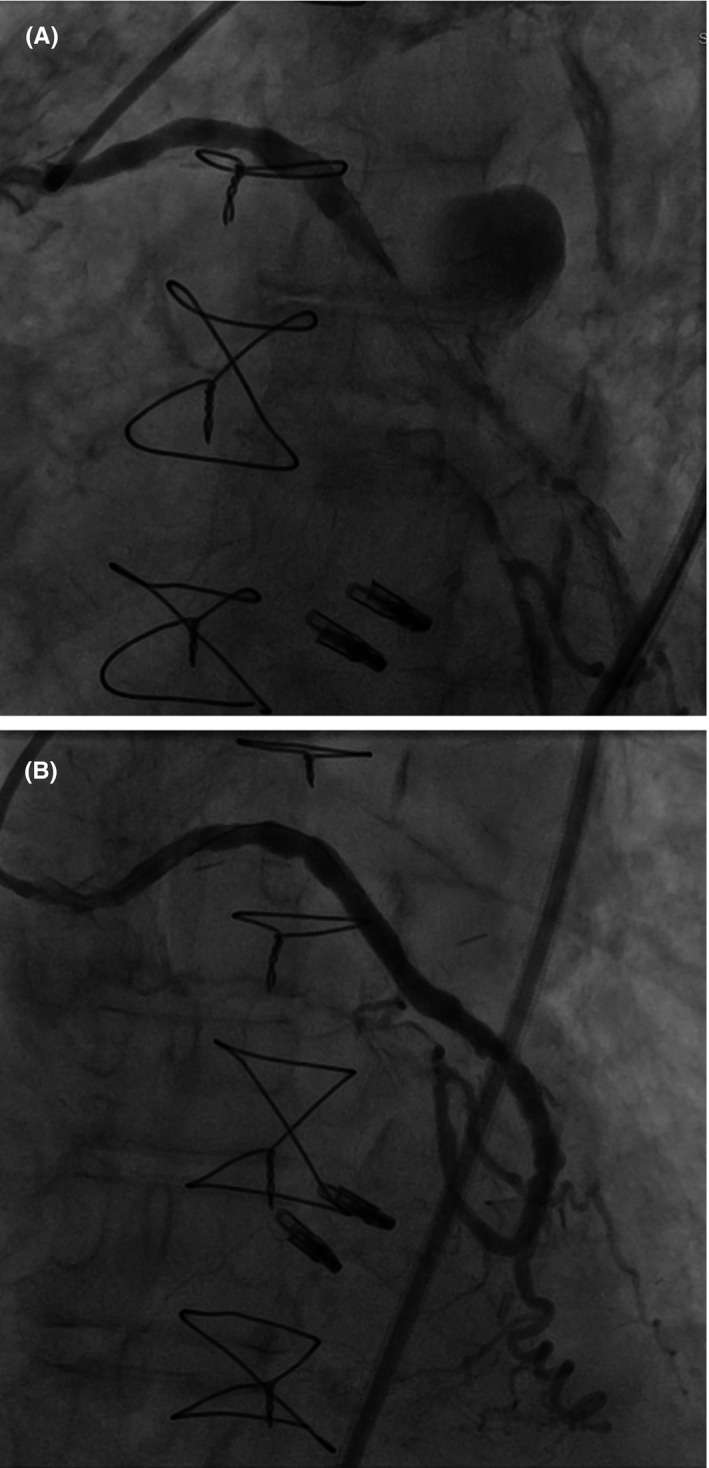
A, Coronary angiogram showing a large aneurysmal sac originating from mid‐segment of body of the SVG to OM. B, Final angiogram showing successful exclusion of the aneurysm along with hemostasis after Papyrus 4.5 × 26 mm stent placement

Diagnostic angiogram showed a large aneurysmal sac originating from the body of SVG (Figure 7A and Video S1). Two GRANDSLAM (Abbott Vascular, Santa Clara, CA) wires were placed in the distal SVG and Eagle‐Eye IVUS (intravascular ultrasound) catheter was then used to assess the reference vessel diameter which was 4.8 mm. One of the GRANDSLAM wires was removed as 0.014” wire compatible stent (Papyrus) was planned to be placed. Next, a 4.5 x 26 mm Papyrus covered stent was deployed at 6 atm with repeat angiogram showing excellent stent expansion along with no residual bleeding and TIMI III flow (Figure 7B, Video S2, Video S3).

Dual antiplatelet therapy was initiated and apixaban was discontinued. A chest tube was placed in left hemithorax draining 2L of frank blood. Thereafter, hemodynamics and serial hemoglobin levels remained stable. The patient was discharged on day‐3 on clopidogrel and apixaban.

## 2 DISCUSSION

Among various postoperative SVG outcomes, saphenous vein graft aneurysmal dilation has a low incidence (0.07%) but with significant clinical consequences.^1^ Aneurysm is defined as >1.5 times focal dilation of the vessel compared to its proximal reference diameter. About 1/3rd of SVGAs are reported to be suspected or confirmed pseudoaneurysms.^2^

Atherosclerotic degeneration of SVGs is thought to be the cause for late graft aneurysmal dilation due to weakening of the vessel wall.^3^ Vessel wall ischemia due to vasa vasorum disruption during harvesting and the grafting process, occult injuries with percutaneous coronary interventions (PCI) or reoperative surgical procedures, exposure to pulsatile arterial pressures, large conduit compared to run off vessel, unfavorable angle at distal touch down, endothelial dysfunction, and changes in conduit wall smooth muscle orientation may initiate a cascade ending up in late SVGA formation.^4^ Contrarily, intrinsic graft wall weakness, direct trauma to the graft during various steps of the procedure, graft infection, and failure to reverse the graft can potentially lead to early SVGA formation. The majority of the patients (68.5%) present after 10 years of CABG procedure, only 4.2% within the first year, favoring atherosclerotic disease as the underlying etiology for this process.^2^ In our case, the SVG was 15 years old with prior PCI with placement of a drug‐eluting stent in the distal segment of the graft >6 years prior.

Although a third of SVGAs are reported as an incidental mass on chest x‐ray, chest pain is the most common (46.4%) presenting symptom followed by shortness of breath (12.9%), myocardial infarction (7.7%), shock (4.3%), hemoptysis (3.8%), and syncope (1.9%).^2^ More than 1/3rd of patients had mechanical complications including cardiac chamber compression (22%), fistulae (7.7%), thoracic vascular compression (7.2%), and aneurysm rupture (8.1%) with hemothorax in 1.8% of the cases with reported 30‐day/in‐hospital mortality of 16.2%.^2^

Diagnostic assessment involves confirmation of the diagnosis, evaluation of the extent of the problem including size and location of the SVGA, mechanical complications, patency of the graft, myocardium supplied by the graft, status of other grafts or native coronaries, additional cardiovascular conditions needing intervention such as valvular abnormalities, procedural risk and feasibility review, and hemodynamic stability. The vast majority of SVGAs are reported to have significant clot burden inside them, hence potentially impact the findings of coronary angiography.^5^ A multimodality approach looking at anatomical details including possible complications and related cardiovascular conditions is needed. Various imaging modalities can be used to assess SVGAs including echocardiography (transthoracic and/or transesophageal), magnetic resonance imaging (MRI/MRA), and simply chest x‐ray, but computed tomography (CT) and coronary angiography are the most commonly used tools in this condition.

In our case, CT scan and coronary angiography were performed during the acute presentation of the patient and SVGA was first suspected on transesophageal echocardiogram performed for other reasons, as an incidental finding followed by confirmation of the diagnosis on CT scan.

CT helps define the anatomy of the SVG aneurysm and its relation to surrounding structures. When active bleeding is suspected, it helps identify the site of bleeding, the size of the aneurysm, any compressions of surrounding mediastinal or cardiac structures, and hence plan therapeutic intervention, that is, open vs endovascular repair. This is especially significant in cases, where prior CT imaging is unavailable. In such cases, it helps define position of the aneurysm in reference to aortic or coronary attachment and neighboring structures. It can also help assess the effect of bleeding aneurysm on neighboring structures. Cardiac MR is a more time‐consuming diagnostic tool and can be of help in stable conditions to better assess anatomy. In many cases, MR angiogram can be suboptimal to assess the vascular details due to respiratory variabilities. Depending upon the expertise of different centers, preference between CT and MR can be variable. Considering acute presentation, CT was the preferred modality by emergency team in this case.

Optimal treatment selection for SVGAs should be based on detailed vascular anatomical features and patient‐related factors. The most prevalent management approach for SVGAs is surgical (>50%), followed by conservative care (20%). Surgical procedures include ligation or resection of the graft followed by placement of a new graft in cases, repair, or placement of interposition graft.^6^ Percutaneous interventional treatment has been reported in around 15% of the cases, includes coil embolization, stent placement, and or vascular plug placement. With newer generation interventional tools including covered stents with easier deliverability, the percutaneous approach offers a lower procedural risk in appropriately selected patients. Uninterrupted lifelong dual antiplatelet therapy may be warranted in cases of covered stent placement. In case of mechanical compression of adjacent structures, not amenable to percutaneous treatment options, surgical treatment can be considered.

Intravascular imaging such as IVUS can be very useful to assess the vessel size for appropriate stent selection. Current coronary covered stents can be used (off‐label) to treat SVGAs with reference vessel diameter up to 5.5 mm and off‐label use of peripheral stents in SVGAs with larger reference vessel diameter. If a 0.035” wire cannot be safely placed in the vessel under treatment, two or three 0.014” wires can be placed in the SVG and used as a railing to deliver an appropriately sized peripheral stent. Based on CT images available, aneurysmal segment was in the body of the graft with a good proximal and distal reference vessel for appropriately sized stent (landing zones). The size of stent needed was more of a dynamic decision process. Considering the emergent nature of the procedure, two supportive coronary wires were placed in case needed as railing for a peripheral covered stent in a large bore saphenous graft. However, Eagle‐Eye IVUS (intra vascular ultrasound) catheter used revealed a reference vessel diameter of 4.8 mm, compatible with a coronary covered stent.

As noted in peripheral the vascular bed, SVGAs may grow in size over a period of just 6 months.^7^ The overall risk of complications (myocardial infarction, rupture, mechanical compression of adjacent structures, and death) rises with increase in SVGA size (33.3% for ≤ 20 mm to 69.2% for > 100 mm).^2^ This cutoff is better studied in peripheral arterial aneurysms but observational data on saphenous vein grafts also goes along the same pattern. Any aneurysm smaller than 2 cm can be closely monitored and managed conservatively, with the knowledge of possible bleeding complications even with this size. Risk of complication is significantly higher once the aneurysm grows bigger than this. Interestingly, the risk for myocardial infarction (~20%) is noted to be higher for smaller size SVGAs (≤20 mm) compared to larger ones (likely due to easier washout of smaller aneurysmal sacs by blood jets leading to distal clot embolization.^2^ Increased overall complication risk with larger size SVGAs is mainly driven by mechanical compression of the adjacent structures rather than rupture or myocardial infarction. Hence, defining a safe size cutoff for surveillance versus early intervention is difficult. These cases should be discussed in a heart team meeting such as structural heart team, to offer customized management. An appropriate follow‐up is warranted for these patients based on their individual risks because recurrence of SVGAs around the stent or in a different area along the length of the graft has been reported.

## 3 CONCLUSION

A multi‐specialty heart team approach with prompt diagnosis, imaging, and treatment in acute presentation of SVGA leads to favorable outcomes.

## CONFLICT OF INTEREST

All authors have declared they have no conflicts of interest relevant to this manuscript.

## AUTHOR CONTRIBUTIONS

Eric Gnall: drafted the manuscript, the main conceptual ideas, and proof outline. Manju Bengaluru Jayanna: careful editorial and technical review, critical revisions of the manuscript. Timothy Shapiro: careful editorial review, provided critical feedback, and helped shape the manuscript. Muhammad Abdulbasit and Maria Elena Drosou: careful editorial and critical revisions of the manuscript. Tariq Ahmad: drafted the manuscript, the main conceptual ideas, supervised the work and as the corresponding and as the last author, is the main person responsible for the contents of the article. All authors discussed, contributed, and approved the final manuscript.

## ETHICAL APPROVAL

As a case report, this work was exempt from need for IRB approval.

## Supporting information

Video S1

Video S2

Video S3

Supplementary Material

## Data Availability

Data sharing not applicable to this article as no datasets were generated or analyzed during the current study.
