# Supplementary material for: “Water running in my chest”: Delayed spontaneous rupture of an aortocoronary saphenous vein graft aneurysm
Source: Clin Case Rep. 2021 Mar 4;9(4):2317–22. doi: 10.1002/ccr3.4024 (PMC8077387; doi:10.1002/ccr3.4024)
Supplement: Supplementary file 4 — Supplementary Material [file CCR3-9-2317-s004.docx]

**Video 1**

Coronary angiogram showing a large aneurysmal sac originating from mid-segment of body of the SVG to OM

**Video 2**

Coronary angiogram showing successful Papyrus 4.5 x 26 mm stent placement.

**Video 3**

Final coronary angiogram showing successful exclusion of the aneurysm along with hemostasis.
